# Description of the EUROBIS Program: A Combination of an Epode Community-Based and a Clinical Care Intervention to Improve the Lifestyles of Children and Adolescents with Overweight or Obesity

**DOI:** 10.1155/2014/546262

**Published:** 2014-08-04

**Authors:** Claudia Mazzeschi, Chiara Pazzagli, Loredana Laghezza, Dalila Battistini, Elisa Reginato, Chiara Perrone, Claudia Ranucci, Cristina Fatone, Roberto Pippi, Maria Donata Giaimo, Alberto Verrotti, Giovanni De Giorgi, Pierpaolo De Feo

**Affiliations:** ^1^Healthy Lifestyle Institute, Centro Universitario Ricerca Interdipartimentale Attività Motoria (C.U.R.I.A.MO.), University of Perugia, Via Giuseppe Bambagioni 19, 06126 Perugia, Italy; ^2^Department of Psychiatry, Clinical Psychology and Psychiatric Rehabilitation, Specialty School of Psychiatry, University of Perugia, Piazzale Gambuli, 1-06132 Perugia, Italy; ^3^Department of Health Prevention, Umbria Region, Via M. Angeloni, 61-06124 Perugia, Italy; ^4^Department of Medicine, Pediatric Clinic, University of Perugia, Piazzale Menghini, 1-0612 Perugia, Italy

## Abstract

The present paper describes the Epode Umbria Region Obesity Prevention Study (EUROBIS) and aims to implement the C.U.R.I.A.MO. model through the EPODE methodology. The main goal of the EUROBIS is to change the pendency of slope of the actual trend towards the increase in the yearly rates of childhood overweight and obesity in Umbria and to improve healthy lifestyles of children and their parents. The project is the first EPODE program to be performed in Italy. The aims of the Italian EUROBIS study are: (1) a community-based intervention program (CBP) carrying out activities in all primary schools of the Umbria Region and family settings as first step, to reverse the current obesity trend on a long-term basis, and (2) a clinical care program for childhood and adolescent by C.U.R.I.A.MO. model. C.U.R.I.A.MO. model is a multidisciplinary approach to improve three key aspects of healthy lifestyles: nutrition, exercise, and psychological aspects with the strategy of a family-based approach. The community-based intervention and clinical trial provide an innovative valuable model to address the childhood obesity prevention and treatment in Italy.

## 1. Introduction

The prevalence of overweight and obesity in children and adolescents is increasing rapidly with dramatic consequences for health [1]. A study of the prevalence and determinants of pediatric overweight and obesity in European countries reveals that the highest values are found in Italy. Italian boys and girls show higher age-specific values of body mass index (BMI), body circumference, waist/hip, and waist/height ratios when compared with other countries [2]. In Italy, pediatric obesity is one of the major public health emergencies: 25% of subjects aged between 0 and 18 years (average) are overweight, with a peak recorded in the 9- to 11-year age group, in which 23% of the population is overweight and 13% is obese. Specifically, the Umbria Region has a prevalence of overweight (26%) and obesity (9%) above the national mean [3].

Pediatric obesity is a complex phenomenon. Its development and maintenance are influenced by a complex array of factors, genetic predisposition, metabolic and neurobiological factors with lifestyle aspects, eating and physical activity habits, and psychological-psychosocial factors [4–6]. Although genetic and biological risk factors are receiving significant research attention, among psychosocial factors in the last decade there has been an important shift by considering individual factors, to focus on environmental factors, given the evidenced systemic associations between adiposity and familial and parental functioning [7]. Obesity runs in families [8] and a series of familial variables, connected to the multifactorial nature of children overweight, have been identified [9–13]. There are evidences that lifestyle behaviors have their roots early in life and recent studies emphasize the impact of parental and familial variables as risk factors on the development and maintenance of childhood overweight and obesity [14].

The purpose of this paper is to describe the Epode Umbria Region Obesity Prevention Study (EUROBIS), an innovative program based on a community-based approach (EPODE,* Ensemble Prévenons l'Obésité des Enfants*) combined with a clinical intervention in order to prevent and treat overweight and obesity in childhood. EUROBIS is twofold: (1) it is to be intended as a community-based intervention program (CBP) to reduce childhood obesity prevalence carrying out activities in all primary schools of the Umbria Region and family settings as first step, and (2) it has a clinical care program by the mean of C.U.R.I.A.MO. (Centro Universitario Ricerca Interdipartimentale Attività Motoria) model to treat childhood and adolescence overweight and obesity through a family-based approach. The model is based on a multidisciplinary approach, already experimented for adulthood obesity [15–17] and tailored for developmental needs. The present paper describes a strategy that combines a strong prevention approach with a strong management approach. Combining these kinds of interventions into one program is very important and innovative and if successful could mean a breakthrough in combating the obesity epidemic.

## 2. Study Design: The Combination of Preventive and Curative Action

EUROBIS is one of the EPODE programs. EPODE is a coordinated, capacity-building approach that aims to reduce childhood obesity through a social process in which local environment, childhood settings, and family norms are directed and encouraged to facilitate the adoption of a healthy lifestyle in children [18].

Within the framework of the EPODE methodology, in Italy EUROBIS is based on a combination of preventive and curative strategies. Relying on the preexisting Healthy Lifestyle Institute of the region, EUROBIS aims to overcome the division between prevention and health care.

In accordance with the EPODE philosophy, it is based on multiple components, including a positive approach in tackling obesity, with no cultural or social stigmatization; step-by-step learning; and an experience of healthy lifestyle habits, tailored to the needs of all socioeconomic groups. EPODE target groups are children, families, local stakeholders, and decision makers working in the different sectors of the society involved in environment causes and determinants of childhood obesity. The four EPODE pillars (political commitment, social marketing, mobilization of resources including public-private partnerships, and evidence including a multidisciplinary evaluation) have been subdivided into ten EPODE implementation principles, which describe the EPODE methodology.

1. Each country (or region) commits to a central coordination support/capacity.
2. Each local community has a formal political commitment for several years from the outset.
3. Each local community has a dedicated local project manager with sufficient capacity and cross-sectoral mandate for action.
4. A multistakeholder approach is integral to the central and local structures and processes.
5. An approach to action is planned and coordinated using social marketing. This is specifically to define a series of themed messages and actions, informed by evidence, from a wide variety of sources, and in line with official recommendations.
6. Local stakeholders are involved in the planning processes and are trusted with sufficient flexibility to adapt actions to local context.
7. The “right message” is defined for the whole community. However, getting the message “right” means tailoring for different stakeholders and audiences.
8. Messages and actions are solution oriented and designed to motivate positive changes and not to stigmatize any culture or behaviors.
9. Strategies and support services are designed to be sustainable and backed by policies and environmental changes.
10. Evaluation and monitoring are implemented at various levels. This is achieved through the collection of information on process, output, and outcome indicators and informs the future development of the program.

An approach to action is planned and coordinated using social marketing. Local stakeholders are involved in the planning processes. Strategies and support services are designed to be sustainable and backed by policies and environmental changes. Evaluation and monitoring are implemented at various levels. EUROBIS, similar to the Epode-like program in the Netherlands (JOGG) (http://www.epode-international-network.com/programmes/jogg), has added a fifth pillar to the ones of EPODE methodology, “linking prevention and healthcare,” and proposes a structured combination of preventive and curative actions. In line with the EPODE methodology to mobilize local resources, the added fifth pillar concerning mental health refers to the Healthy Lifestyle Institute of Perugia University. The model is based on a multidisciplinary approach, already experimented for adulthood obesity [15–17, 19], tailored for children and adolescents. The intervention program differs for children and adolescents and it is finalized in improving three key aspects of healthy lifestyle: nutrition, exercise, and psychological wellbeing, using a family-based approach. The C.U.R.I.A.MO. model for pediatric overweight or obesity involves the following health care professionals: pediatricians, endocrinologists, psychologists, dieticians, and exercise physiologists.

**Figure 1:**
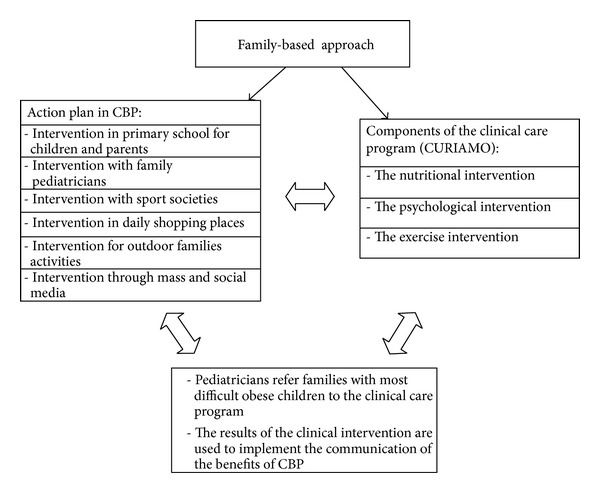
Diagram describing the action plan of the community-based program (CBP) and the clinical care program (C.U.R.I.A.MO.) and the interaction between the preventive and the curative strategies.

As illustrated in Figure 1, the pediatricians have a key role between the two approaches (CBP and C.U.R.I.A.MO.) both facilitating families with overweight or obese children in referring to the clinical care program and communicating the results of the clinical intervention in order to implement the benefits of CBP.

A common perspective of both CBP and C.U.R.I.A.MO. is the family-based approach with an active involvement of the parents in the project of changing. The main aim is to actively involve both the child/adolescent and the two parents (as far as possible) in order to mobilize family resources to improve the efficacy of the program. In this model, the objective is to involve the parents in order to improve their skills and confidence. Working with general and specific parental skills connected to child's care and health, it is possible to teach the parents to recognize the child's needs, manage child's dietary and activity patterns, and promote a healthy lifestyle in the family and consequently in the child. In adolescence it is also necessary to improve parental skills in recognizing the impact of overweight/obesity in the self-esteem of the youth.

The characteristics of CBP and C.U.R.I.A.MO. models will be discussed in detail separately, outlining evaluation and monitoring strategies.

## 3. The Community-Based Intervention Program (CBP)

In order to mobilize stakeholders at all levels across the public and private sectors through a local steering committee and local networks, the Umbria Region (the President and the vice-President of Umbria Region and the regional assessors of health, welfare, and agriculture and local food), the Director of the Health Prevention Department, the local university, family pediatricians, local private associations, the Regional Olympic Committee, the Regional Federation of Industries, promoters of treks and urban walking activities, the major local companies of food distribution, vending machines, the regional media channel, and web journal all actively collaborate with EUROBIS.

### 3.1. Action Plan in CBP

According to the Council Recommendations of the European Commission (http://ec.europa.eu/sport/library/documents/c1/com-2013-603-final-council-recommendation-hepa_en.pdf), the plan of action covers different sectors (health, sport, school, and environment) in order to include a series of actions. In order to promote a healthy lifestyle among children and their families, the actions planned include many contexts:

- intervention in primary school for children and parents: active transport (pedibus); exercise classes; monthly meetings with parents on healthy nutrition and psychological determinants of obesity (production of media books, web sites, and printed materials);
- intervention with family pediatricians: periodical meetings on effective strategies to prevent childhood obesity;
- intervention with sport societies: promotion of baby and child participation in sports independently of their talent, encouraged through award for excellence to the sport societies, and link of these societies to family pediatricians;
- intervention in daily shopping places: health lifestyle corner and healthy cooking classes in major food shops and distribution of healthy food and beverages in vending machines;
- intervention for families: visits to farms and vegetable gardens with tasting on site, mapping of regional healthy trails and promotion of open air activities for families;
- intervention through mass media: global media communication strategy to fight childhood obesity including campaigns to promote healthy nutrition and regular exercise with a family-based approach using a web site (http://www.eurobis.it) and social channels (Facebook, Twitter, YouTube, and Google+).

### 3.2. Evaluation and Monitoring of CBP

The overall expected outcome for the community-based intervention is to change the pendency of the slope of the actual trend towards the increase in the yearly rates of childhood overweight and obesity in Umbria. Umbria Region has an efficient system of surveillance for the epidemiology of overweight and obesity in children by means of three different approaches: (1) anthropometric measurements performed by family pediatricians every 5 years; (2) a survey “OKKIO alla Salute” (http://www.iss.it/binary/publ/cont/0924.pdf), of a significant number of families from Umbria every 2 years; and (3) a survey Studio PASSI (http://www.iss.it/binary/publ/cont/07-30.1195128446.pdf) of a significant number of families from Umbria every 2 years.

Therefore a historical epidemiology database on overweight and obesity in children from Umbria is available and can be used as a baseline. It is also possible to make comparisons with the closest Regions, which have a lower prevalence of childhood obesity than in Umbria. The process will be monitored and evaluated by measuring in significant subgroups of children body composition, with noninvasive techniques (Bod Pod: air displacement methodology). Continuous monitoring and evaluation practices at a local level will regard input, activities, output, and outcome indicators. The evaluation process will take into account the participation of intervening parties, awareness raised among the political representatives involved in the program, local stakeholders' feeling as part of a common positive action for the community, and participation of the families and children in the program's activities.

A program efficacy indicator will be the number of EUROBIS actions that will be adopted by the 2014–2018 Umbria regional health prevention program. The process of evaluation will be performed by the* Steering and Scientific Committees* examining the data of interviews and questionnaires periodically administered to the target population (children and their parents) of EUROBIS. The monitoring and evaluation also consist in data collection, performed by health professionals, on weight, height (BMI measurements), and waist circumference of children. Other indicators will include physical child performance, energy expenditure (METS/h/week) during leisure time, number of meals consumed in family and quality of the child's life (reported by parents), indicators of well-being both from parents and child perspective, level of healthy attitudes, health status and level of participation of the child in daily life activities (inside and outside families) [20]. Measurements of the interventions impact and publication of the results in international scientific journals, production of media books, web site and printed materials will contribute to the dissemination of the program results.

## 4. The Clinical Care Program

*Study Design*. Over three years about 90 children (aged 5–10 years) and 90 adolescents (aged 11–16 years) with overweight or obesity will be enrolled. Inclusion criteria will be as follows: age between 5 and 16 years; BMI higher than 90% percentile. Exclusion criteria will be concomitant diseases contraindicating physical exercise. The enrollment is planned to include 30 subjects every 6 months and to perform the lifestyle intervention described below, in order to reach a total number of about 60 patients/year. The total duration of the study will be 4 years. The medical examination performed by the pediatrician in our institute is finalized to establish the degree of overweight or obesity, the absence of diseases responsible of obesity, and the lack of contraindications to physical exercise.

### 4.1. The Three Components of the Clinical Care Program

*The Nutritional Intervention.* The aim is to train children's parents or directly adolescent patients (11–16 years) to be able to regularly choose and eat healthy foods. The intervention is structured in two counseling sessions with a dietician (30 minute of duration at 1 month interval) with children's parents or directly with the adolescent patients and in four educational group sessions. During the two counseling sessions, for promoting weight loss, the dietician does not prescribe a restricted diet but provides nutritional information and uses food log for monitoring dietary habits and their changes. The four nutritional education group sessions are conducted by two dieticians; they last about 120 minutes and are based on interactive learning. Twelve to sixteen parents (both mothers and fathers) of children (5–10 years old) are invited to the four educational sessions or, in the case of adolescents (11–16 years old), directly 6–8 patients are invited, while their parents are allowed to attend only the first of the four sessions, dedicated to elucidate the general principles of a healthy nutrition. During the educational sessions the dieticians interactively illustrate the strategies to reduce high energy density food consumption in order to cut daily caloric intake of about 300–400 Kcal and daily caloric intake from fat to <30% of total caloric intake (ideal composition of diet: CHO 55%, FAT 30%, and protein 12–15% of total calories) and the strategies to increase the Mediterranean diet score by eating more frequently vegetables, fish, fruit, and food naturally rich in fibers. Every 3 months, in occasion of maintenance nutritional visits, patients' nutrient intake is monitored by food logs reported by children's parents or by adolescent. In order to estimate the changes in nutritional habits the children's parents or the adolescent patients will fill a validated questionnaire to calculate the Mediterranean diet score [21].

*The Psychological Intervention.* The intervention is primarily characterized by counseling centered on each family's needs, by assessing their characteristics, their strengths, and weaknesses in different domains.

- Anamnestic, regarding the child and his parents: there is evidence on the relation between some traumatic life events and obesity [22].
- Personal, regarding each parent separately and the child: this domain refers specifically to anthropometric and psychological risk factors identified as associated with child's obesity. Among them is parents' BMI considered separately, as contributing differently to child's BMI [23], maternal depression, or anxiety [12, 24].
- Familial, considering the family as whole and its functioning in terms of structural factors and emotional climate: family socioeconomic level (SES) has been identified as being correlated with child overweight [10] as well as number of siblings in the family, order of birth, and type of family (in marriage, alone mothers, or alone fathers).
- Parental, from both parents' perspectives (as far as possible): their contribution to the child's care, their parental practice, including modeling [25], their way of caring for the child in terms of style of parenting [10, 26], their alliance in managing the child's development, and the level of distress.

The aim is to create, during the assessment-intervention phase, a tailored psychological program based on each family and parental characteristics along with a shared program followed by the children/adolescent and parents.

The psychological work follows three steps: (1) a first step of psychological assessment; (2) a second step of psychoeducational groups of parents (a) or of adolescents (b); and (3) and (4) two follow-up sessions.

*Step 1 (psychological assessment)*. The program starts with an initial intensive phase (five sessions lasting one month and a half), with the child/adolescent as well as with the parents in order to assess, through the use of psychometric psychological measures, a series of risk factors associated with overweight/obesity in families (anamnestic, personal, parental, and familial) and to assess the presence of eating disorders or other psychological complications according to the guidelines of the Italian Society of Obesity.

*Step 2a (psychoeducational group of parents (four sessions))*. Both parents are invited to participate in four sessions in small psychoeducational groups (eight to ten people) in order to sustain the child's physical and nutritional program. By using video-recorded material, consisting of scenes derived by a series of movies identified as evocative of specific triggering familial and interactional episodes (POR FSE 2007–2013, Umbria Region Grant 2011/2012), parents (mothers and fathers) participate in a series of psychoeducational sessions. By using video-feedback and self-reflection, parents are trained to allow more insight and empathy, broadening their coping skills toward the use of healthy life habits.

*Step 2b (psychoeducational peer group of adolescents (three sessions))*. Adolescents are invited to participate in a three-session psychoeducational peer group on the impact of overweight and obesity on self-esteem and on body image. The aim of the group is to share with peers, with the help of a psychologist, the major risk factors of obesity associated with this developmental age.

*Steps 3 and* *4 (first followup at 6 months and second followup at one year)*. Clinical interview and psychometric evaluation are conducted for parents and children/adolescents separately, in order to assess program-related changes over the course of treatment and at the end of it. Followup is conducted according to a collaborative approach.

*The Exercise Intervention.* The exercise protocol intervention is planned with duration of 6 months and a frequency of 2 sessions/week and it is different for children (5–10 years old) and adolescents (11–16 years).

The exercise program for children aims to achieve two outcomes: (a) to improve general physical fitness including spatial coordination, aerobic capacity, flexibility, and muscle strength; (b) to promote the discovery of the feeling of fun with traditional games based on active motion. The activities programmed are performed in the gym for groups of 6–8 kids supervised by exercise physiologist and they consist of four phases: warm-up, exercises for general body fitness, game group, and final stretching for a total of 70–80 minutes per session. During the first and the last of the 52 sessions functional tests are performed to evaluate the aerobic capacity, flexibility, and dynamic muscle strength [27–32].

The training program for adolescents is performed in the gym and supervised by an exercise physiologist with a maximum attendance of 5 patients/group. Each session lasts 90 minutes divided into 60 minutes of aerobic workout and 30 minutes of circuit training for muscular strength and flexibility exercises. The aerobic workout is performed using ergometers for cardiovascular work (treadmill, cicloergometer, and armergometer) with gradually increasing intensity of work up to 60–70% of heart rate reserve. The workout for muscular strength will use machines and isotonic free loads for training the lower and upper limbs, with gradual increase up to 70–80% of 1 repetition maximum (RM). During the first and the last session aerobic capacity and muscle strength will be measured. Aerobic capacity is estimated using the Rockport Fitness Test [27] on treadmill. The determination of the maximum dynamic force [28, 29] of extensor muscles of the leg and the flexor and extensor muscles of the arms is conducted by the indirect method of extrapolation to one repetition max by using MRI leg press, lat machine, and chest press machine (Technogym, Cesena, Italy).

### 4.2. Evaluation and Monitoring of Clinical Care Intervention

The aim is to improve the adoption of a healthy lifestyle by children and adolescents. The family-based approach includes the parents as targets of the intervention for nutritional education and the psychological support; in addition for overweight adults there will be the possibility to participate in supervised exercise sessions, during the physical training of their children, using the C.U.R.I.A.MO. model (15). The major outcome of the population intervention will be a reduction of at least 5% in BMI and waist circumference percentiles in children from Umbria and adolescents after 4 years of intervention. The primary outcome is an improvement in lifestyle, using a composite end-point. We postulate that at the end of the study (4 years) more than 70% of children and adolescents enrolled in the C.U.R.I.A.MO. project will improve their lifestyle. Multiple imputations for missing data and intention-to-treat analysis will be used for statistical purpose. A significant improvement in lifestyle (composite major end-point) is defined as an increase of at least 20% of the Mediterranean diet index score combined with an increase of at least 10 MET/h^−1^·week^−1^ of energy expenditure by physical activity and a reduction of at least 5% of the percentiles of BMI and/or waist circumference. Measures of quality of life and psychological well-being will also be included both from parents and children perspectives, aimed at assessing the increased level of healthy attitudes, the level of health status (considered in a broad sense), and the level of child participation in daily life activities (inside and outside families) [33].

## 5. Discussion

The American Academy of Pediatrics Expert Committee Recommendations Regarding the Prevention, Assessment, and Treatment of Child and Adolescent Overweight and Obesity [34] suggested a family-based approach to treat pediatric obesity. According to Kitzman and Beech, family-based interventions are defined as active parent involvement in treatment [35]. There are evidences of clear advantages associated with family-based intervention. Family-based approach is the “gold standard” [36] for pediatric obesity treatment, showing the strongest and longest lasting effects with the inclusion of parents [37, 38]. The approach focused on parents reflects the recognized multifactorial nature of pediatric obesity, engaging both genetics and environmental factors [13] and the fact that lifestyle aspects are consistently shown to be highly predictive and can be more changeable by treatment interventions [5].

Parent self-report measures of adherence outside the treatment setting have been identified as better predictors of child outcome than objective measures: family adherence to the treatment protocol has been identified as a good predictor of treatment success [39]. This conclusion has been reached also by Yackobovitch-Gavan and colleagues devising a major reduction of BMI in those children whose parents completed self-reported measure before and after treatment [24]. Gilles et al. found that increasing parental involvement expands the rate of success [40]. Moreover, parental functioning influences the course of the treatment [41, 42]. In this direction there are studies showing that in pediatric obesity treatment caregiver parental distress is an influential factor compromising successful outcomes and that children's perception of father's acceptance of their treatment is an important factor for a greater weight loss [26, 43, 44]. Zeller and colleagues identified in caregivers of youth treatment seeking for obesity a greater psychological distress, more family conflict, and greater mealtime challenges compared to caregivers of youth of healthy weight [41]. Moreover, in caregivers of school-aged children a high percentage of clinically elevated levels of spousal discord specific to parenting has been detected [12]. Lower maternal sensitivity, measured by direct observation of parent-adolescent interactions, was found to be associated with adolescent obesity [45]. According to Epstein and Wrotniak, there is need to develop new paradigms to treat pediatric obesity by devising programs based on moderators of treatment success to be translated into clinical interventions [39].

Starting from this perspective, the aim of the present study was to illustrate an innovative model trialled by public and private sectors to promote an active and healthy lifestyle in childhood and adolescence overweight and obesity. The model proposed, the Italian EUROBIS study based on an EPODE methodology, is twofold. Both the community-based intervention program and the clinical care program, carried out by the means of the C.U.R.I.A.MO. model, aim to involve parents and their children in the prevention and treatment of overweight and obesity. In order to achieve this aim, the actions planned in the community-based intervention are primarily directed toward involving the whole family. The clinical care program is based on the C.U.R.I.A.MO. model, chosen to entirely counteract the three main factors involved in the current rise of pediatric obesity by enhancing (1) physical activity, (2) healthy nutrition, and (3) motivation for a correct style of life, by working with parents in order to reduce their impact of unhealthy habits on child healthy behaviors and beliefs and, with adolescents, by promoting their skills and empowerment towards a healthy style of life. In our opinion the multidisciplinary approach that characterizes the model and its family-based approach should lead to the future validation of the intervention.

In conclusion, EUROBIS will explore the efficacy of combining clinical care with CBP. We are confident that, on the basis of the positive results of previous EPODE programs [18], the global strategy adopted in designing EUROBIS intervention will have a significant impact on reducing the burden of childhood and adolescence overweight and obesity in the Region of Umbria.
